# Cannabidiol Rescues Acute Hepatic Toxicity and Seizure Induced by Cocaine

**DOI:** 10.1155/2015/523418

**Published:** 2015-04-27

**Authors:** Luciano Rezende Vilela, Lindisley Ferreira Gomides, Bruna Araújo David, Maísa Mota Antunes, Ariane Barros Diniz, Fabrício de Araújo Moreira, Gustavo Batista Menezes

**Affiliations:** ^1^Laboratório de Imunobiofotônica, Departamento de Morfologia, Instituto de Ciências Biológicas, Universidade Federal de Minas Gerais, Avenida Presidente Antônio Carlos 6627, 31270-901 Belo Horizonte, MG, Brazil; ^2^Departamento de Farmacologia, Instituto de Ciências Biológicas, Universidade Federal de Minas Gerais, Avenida Presidente Antônio Carlos 6627, 31270-901 Belo Horizonte, MG, Brazil

## Abstract

Cocaine is a commonly abused illicit drug that causes significant morbidity and mortality. The most severe and common complications are seizures, ischemic strokes, myocardial infarction, and acute liver injury. Here, we demonstrated that acute cocaine intoxication promoted seizure along with acute liver damage in mice, with intense inflammatory infiltrate. Considering the protective role of the endocannabinoid system against cell toxicity, we hypothesized that treatment with an anandamide hydrolysis inhibitor, URB597, or with a phytocannabinoid, cannabidiol (CBD), protects against cocaine toxicity. URB597 (1.0 mg/kg) abolished cocaine-induced seizure, yet it did not protect against acute liver injury. Using confocal liver intravital microscopy, we observed that CBD (30 mg/kg) reduced acute liver inflammation and damage induced by cocaine and prevented associated seizure. Additionally, we showed that previous liver damage induced by another hepatotoxic drug (acetaminophen) increased seizure and lethality induced by cocaine intoxication, linking hepatotoxicity to seizure dynamics. These findings suggest that activation of cannabinoid system may have protective actions on both liver and brain induced by cocaine, minimizing inflammatory injury promoted by cocaine, supporting its further clinical application in the treatment of cocaine abuse.

## 1. Introduction

Drug abuse and addiction constitute a public health problem of great importance with a high prevalence worldwide. According to the United Nations Office on Drugs and Crime (UNODC), cocaine, a psychostimulant and psychotomimetic drug, is among the most abused drugs in the world. The estimated number of cocaine users globally ranges between 14 and 21 million (0.3–0.5% of the population aged between 15 and 64 years) [1]. Besides its toxicity for the cardiovascular central nervous systems, cocaine causes liver injury in human and animal models [2, 3]. This drug may lead to severe acute hepatotoxicity due to hepatocellular necrosis, which can be life threatening. Indeed, as diminished liver function contributes to various adverse health effects, hepatotoxicity has been linked to the mortality in cocaine abusers [4].

Oxidative stress (OS) plays a key role in cocaine-induced hepatotoxicity and is well reported in both humans and animal models [5]. Furthermore, several lines of evidence indicate that excessive OS with increased free radical generation, impairment of mitochondrial respiration, and intense inflammatory reaction plays a crucial role in cocaine-induced hepatotoxicity. OS is associated with the dysregulation of signaling pathways modulating cell death and survival, which leads to necrosis and apoptosis of hepatocytes [6]. Although previous studies have yielded mechanistic information on cocaine-induced hepatotoxicity, the current understanding on this toxic event remains insufficient [7]. Cocaine users have altered levels of proinflammatory cytokines and chemokines in the plasma, indicating that peripheral immune system adaptations may contribute to the effects of cocaine [8, 9].

It is well described that the prevention of OS generation might have therapeutic advantages in several diseases [6, 10, 11]; therefore, the investigation of putative new compounds with antioxidant properties might be promising. The herb* Cannabis sativa* (“marijuana,” “hemp”) has been known for centuries due to its abuse-related effects and possible therapeutic uses, including the treatment of inflammation, diabetes, cancer, affective or neurodegenerative diseases, and epilepsy-related disorders. Among more than 60 chemical compounds already characterized from this herb, the two most investigated are Δ^9^-tetrahydrocannabinol (Δ^9^-THC) and cannabidiol (CBD) [12–14]. Δ^9^-THC, which accounts for most of the typical cannabis actions, may have therapeutic efficacy against several diseases, although its use is limited by its psychoactive properties. Thus, an alternative for developing cannabis-based medicines could be focusing on CBD, the major nonpsychotomimetic phytocannabinoid. This compound acts through multiple pharmacological targets, including facilitation of the endocannabinoid system, activation of transient receptor potential vanilloid type-1 (TRPV1) channel, the peroxisome proliferator-activated receptor *γ* (PPAR*γ*), GPR55, 5-hydroxytryptamine receptor subtype 1A (5-HT_1A_), the adenosine membrane transporter phospholipase A_2_, lipoxygenase (LOX) and cyclooxygenase-2 (COX-2) enzymes, and Ca^2+^ homeostasis [15]. This rich pharmacology, associated with the fact that it does not share the psychotomimetic, amnestic, and sedative effects of Δ^9^-THC, results in a wide range of potential therapeutic interest, including the treatment of epilepsies and related disorders [16, 17]. Accordingly, a recent clinical study observed that CBD-enriched cannabis extracts significantly reduced seizure frequency in children with treatment-resistant epilepsy, indicating that it could be used as a strategy when other pharmacological treatments fail [18].

Apart from searching for the therapeutic application of phytocannabinoids, other potential strategy focuses on the endocannabinoid system. This intercellular message system comprises the CB_1_ and CB_2_ cannabinoid receptor, the ligands arachidonoyl ethanolamide (anandamide) and 2-arachidonoyl glycerol, their hydrolyzing enzymes (fatty acid amide hydrolase, FAAH, and monoacyl glycerol lipase, resp.), and their synthesizing machinery [19, 20]. Among its numerous biological functions, the endocannabinoid system has been implicated in protection against deleterious stimuli in various tissues, including the brain [21] and the liver [22, 23]. Previous studies also demonstrated that CBD possesses efficient antioxidant and anti-inflammatory activities against ischemia/reperfusion liver injury in rats [24, 25]. Moreover, CBD restored liver/brain function in a model of hepatic encephalopathy associated with fulminant hepatic failure induced in mice revealing that its effects may result from a combination of its actions in the liver and brain [26]. Here, to investigate if CBD exerts protective by CBD exerts protective effects in a model of seizures and liver damage resulting from cocaine intoxication. We have compared the effects of these phytocannabinoids with the FAAH inhibitor URB597, which facilitate endocannabinoid signaling by selectively increasing anandamide levels. Finally, we investigated if previous hepatic lesion induced by other compounds (acetaminophen) [27–30] would modulate SNC-related cocaine effects.

## 2. Materials and Methods

### 2.1. Mice

Male Swiss mice weighing 20–30 g from the animal facility (Centro de Bioterismo, CEBIO) of UFMG were kept on a 12 h : 12 h dark/light cycle at 22 ± 1°C with free access to food and water throughout the experiment. Each animal was used only once. All experiments were conducted in accordance with the Ethical Committee for Animal Experimentation (CETEA) of the Federal University of Minas Gerais (Universidade Federal de Minas Gerais (UFMG)) and procedures for animal care were previously approved by this organization under Protocol 242/2013.

### 2.2. Drugs

Cannabidiol (30 mg/kg; THC-Pharm, Frankfurt, Germany, and STI-Pharm, Brentwood, UK) was dissolved in physiological saline containing tween-80 at 2%. URB597 (1.0 mg/kg; Tocris) was dissolved in ethanol/cremophor/saline 1 : 1 : 18. Cocaine (75 mg/kg; Merck, Darmstadt, Germany) was dissolved in physiological saline. The solutions were prepared immediately before use and injected via intraperitoneal route in a volume of 10 mL/kg. All doses were chosen based on previously published studies [31–34]. Cannabidiol dose was chosen based on dose-response curves previously established in our group [35–38]. Although most of these studies have focused on the anxiolytic and antipsychotic effects of this compound, the dose range for its antiseizure and antiepileptic effects is in the sane range [39]. Thus, we tested anticonvulsant effect of cannabidiol (30, 60, and 90 mg/kg) 30 minutes before cocaine administration. At dose of 30 mg/kg, behavioral parameters of seizure (latency and duration seizure) are significantly inhibited when compared to doses of 60 and 90 mg/kg (data not shown).

### 2.3. Apparatus and Cocaine-Induced Seizures

The animals were injected intraperitoneally (i.p.) with cocaine (75 mg/kg) and immediately placed in individual chambers (40 cm in diameter with a 50 cm high Plexiglas wall) and observed during 10 min for the onset of behavior seizures and the occurrence of death. Seizure was defined as the occurrence of tail clonus with myoclonic jerks and wild jumping or convulsions with loss of righting reflex. Latency and duration for myoclonic seizure were also measured in the subset of mice that progressed to this state.

### 2.4. Model of Acetaminophen-Induced Liver Injury and Inflammation

Acetaminophen (paracetamol; APAP) was orally administered (600 or 800 mg/kg; Sigma) in male Swiss mice weighing 20–25 g from the animal facility (Centro de Bioterismo, CEBIO) of UFMG after 15 h of fasting [27–30]. Control mice received warm sterile saline as vehicle. After 24 h, mice were anesthetized with a mixture of ketamine (80 mg/kg) and xylazine (15 mg/kg) and killed for serum and liver harvesting. In a separate set of experiments, the survival percentage of APAP-challenged mice was evaluated during 24 h. Neutrophil infiltration into the liver was measured by the myeloperoxidase (MPO) activity assay.

### 2.5. Alanine Aminotransferase Assay (ALT)

The alanine aminotransferase enzyme is present in the cytoplasm of hepatocytes and is highly specific for the liver. The measurement of serum alanine aminotransferase (ALT) is a gold-standard marker of liver damage. To determine the activity of ALT, blood samples were centrifuged and the serum was collected and dosed using a kinetic kit (Bioclin, Brazil) [28, 29].

### 2.6. Liver Histological Analysis

Livers were collected 24 h after induction of ALF and fixed by overnight immersion in 10% buffered formalin. Paraffin-embedded specimens were prepared and 5.0 *μ*m sections were stained with hematoxylin and eosin (H&E) according to a standard protocol. Liver pathology was assessed by an investigator who was blinded to the experimental treatment groups in an Olympus BX51 microscope (Center Valley, PA, USA) [28, 29].

### 2.7. Indocyanine Green (Cardiogreen; ICG)

The ICG standard curve was drawn according to the manufacturer's instructions. Indocyanine green was completely dissolved in distilled water and prepared to a final concentration of 0.1 mg/mL. According to the experimental design, each animal was given a caudal intravenous injection of indocyanine green at 20 mg/kg of body weight. One hour after the ICG injection, a laparotomy was performed and the aorta was cut, followed by isolation of plasma with centrifugation at 7000 rpm for 7 min. Plasma (0.15 mL) was extracted and diluted with saline. Absorbance value of the plasma sample was determined by spectrophotometry determining the absorbance of the plasma sample at 805 nm, which was compared with the value for normal plasma. The standard curve was used to calculate the serum concentration of ICG.

### 2.8. Liver Confocal Intravital Microscopy

Liver microcirculation was imaged as described previously [29, 40]. Briefly, mice were anesthetized with a mixture of ketamine (80 mg/kg) and xylazine (15 mg/kg). The liver was gently pulled out through a laparotomy incision and positioned over a Plexiglas stage for imaging under a confocal inverted microscope (Nikon Eclipse Ti and C2 confocal head). For imaging liver microcirculation and necrosis, mice received endovenously 8 *μ*g of phycoerythrin-labeled anti-PECAM-1 (stains liver sinusoidal endothelial cells; eBiosciences, USA) and 2 *μ*L of the stock solution of Sytox Green (stains extracellular DNA deposits and necrosis; Life Technologies, USA). The liver surface was imaged in a custom made stage that holds the liver in flat position. Fluorophores were excited with 488 and 543 nm lasers line using a 10x objective. All images were generated by Volocity software (6.3; PerkinElmer, USA).

### 2.9. Statistical Analysis

Experimental data analysis was performed with one-way analysis of variance (ANOVA) (Tukey's post hoc test). *P* values < 0.05 were considered statistically significant. All experiments included *n* = 6. Data are presented as mean ± SEM. Fisher's exact probability test was additionally used for specific comparisons between seizure parameters and hepatic functions. Graphs and statistical analysis were performed using Prism 5 (GraphPad software, USA).

## 3. Results 

### 3.1. Cocaine Administration Caused Acute Liver Injury and Inflammation

**Figure 1:**
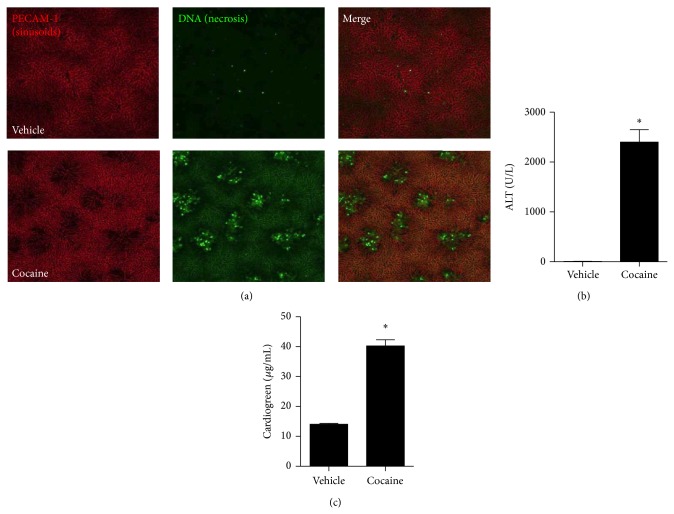
Cocaine intake causes severe liver injury and malfunction. (a) Liver confocal intravital microscopy showing that while control mice have a well perfused liver vasculature (evidenced by PE-anti-CD31 antibody staining, in red) and absence of necrosis (in green), cocaine-treated mice had several dark areas in the red channel, which are suggestive of malperfusion caused by necrosis (elicited by DNA staining using Sytox Green). (b) Serum liver transaminase levels and (c) retention of cardiogreen confirmed necrosis.  ^∗^Statistical significance in comparison to controls (*P* < 0.05). A 10x objective was used during imaging. One-way ANOVA with Tukey's post hoc test. Data are expressed as mean ± S.E.M. (*n* = 6 mice/treatment).

**Figure 2:**
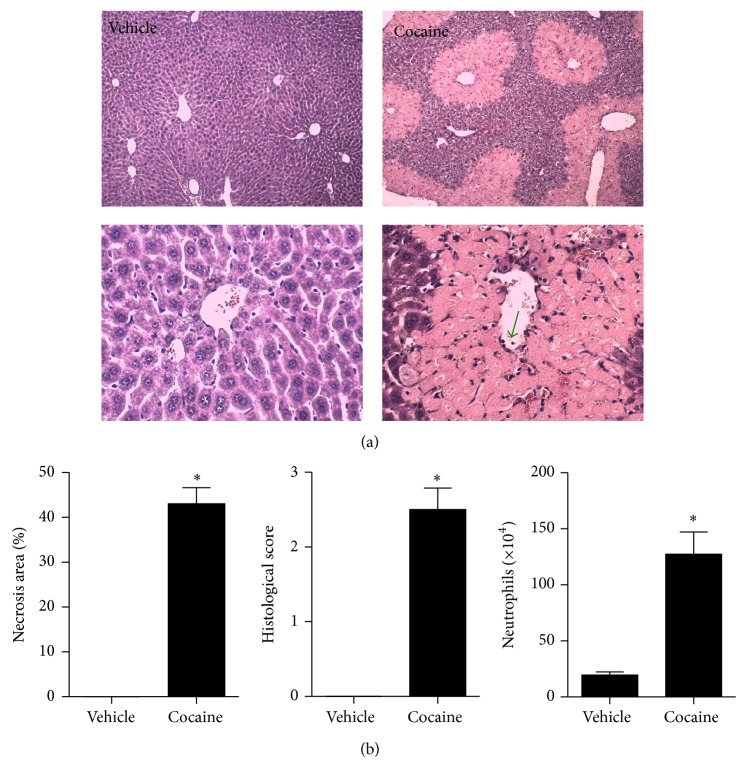
Histopathological assessment of cocaine-induced liver injury. (a) Liver histology (H&E) showing that while control mice have a healthy liver architecture and absence of necrosis, cocaine-treated mice had several necrotic areas with leukocyte infiltration (green arrow). (b) Digital quantification of necrotic areas using ImageJ, histopathological score, and neutrophil infiltration (assessed by MPO levels) confirmed severe liver inflammation and necrosis induced by cocaine.  ^∗^Statistical significance in comparison to controls (*P* < 0.05). A 10x objective was used during imaging. One-way ANOVA with Tukey's post hoc test. Data are expressed as mean ± S.E.M (*n* = 6 mice/treatment).

To investigate the hepatotoxic effects of acute cocaine intake, mice received a single administration of cocaine (75 mg/Kg; i.p.) and livers were imaged under confocal intravital microscopy. As shown in Figure 1(a), control mice had a well-perfused liver microvasculature, as evidenced by sinusoidal staining by anti-CD31 (PECAM-1, in red). Also, intravenous administration of a DNA-binding probe (Sytox green) revealed almost no extracellular DNA under basal conditions. We have previously shown that hepatocytes released DNA into the liver, leading to a widespread hepatic DNA accumulation, which was directly correlated with injury severity and progression. In line with this, mice overdosed with cocaine had massive liver injury, evidenced by lack of perfusion (dark spots on red channel), which were consistently filled by extracellular DNA, linking cocaine intake to acute liver injury. Elevated serum transaminases levels (ALT) confirmed acute hepatotoxicity induced by cocaine (Figure 1(b)). To evaluate if such injury was ultimately impairing liver function, we injected a known amount of indocyanine green (cardiogreen) [41], which is rapidly cleared from the circulation under control conditions by the liver. Interestingly, cocaine-overdosed mice presented a significant retention of cardiogreen in the serum, suggesting that liver metabolic function was also impaired (Figure 1(c)). In fact, liver histopathology showed that cocaine also caused liver leukocyte infiltration together with an extensive necrosis (Figures 2(a) and 2(b)). Together, these data demonstrated that acute cocaine intake led to liver injury and inflammation.

### 3.2. Cocaine-Induced Seizure Is Directly Correlated with Liver Injury

**Figure 3:**
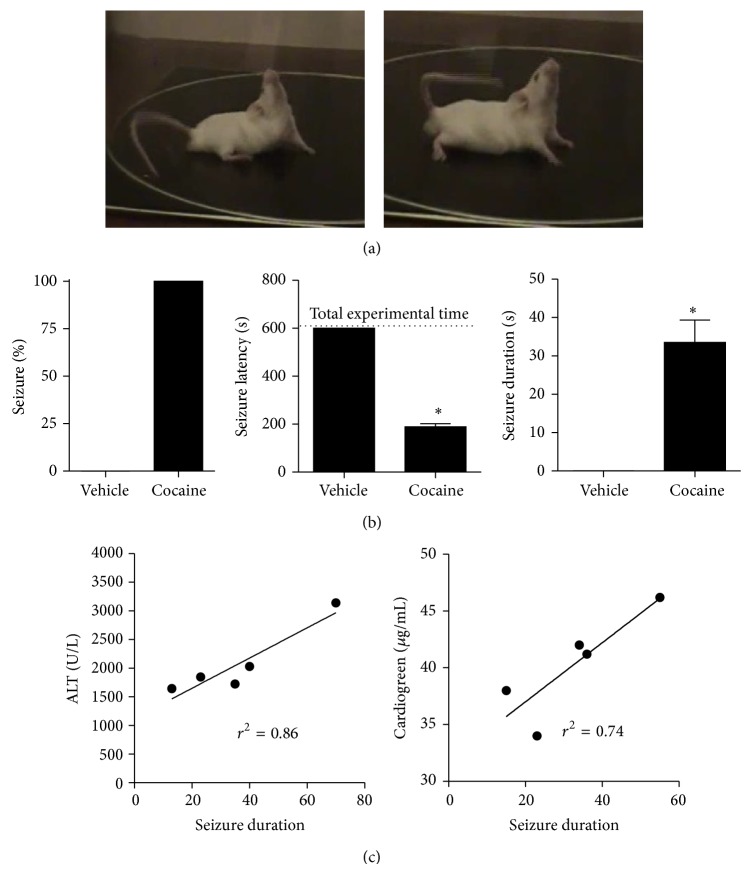
High doses of cocaine promote acute behavioral seizure. (a) Representative picture of seizure characterized by rapid myoclonic convulsions of forelimbs and tail clonus preceded by locomotor depression. (b) Acute overdose induced seizure in all animals represented by latency reduced and duration increased compared to group vehicle. (c) Positive correlation between seizure duration and acute hepatotoxicity induced by cocaine (*r* ^2^ = 0.86, *P* < 0.02 for ALT; *r* ^2^ = 0.74, *P* < 0.05 for cardiogreen).  ^∗^Significantly different from vehicle group (^∗^ *P* < 0.001). One-way ANOVA with Tukey's post hoc test. Data are expressed as mean ± S.E.M (*n* = 6 mice/treatment).

**Figure 4:**
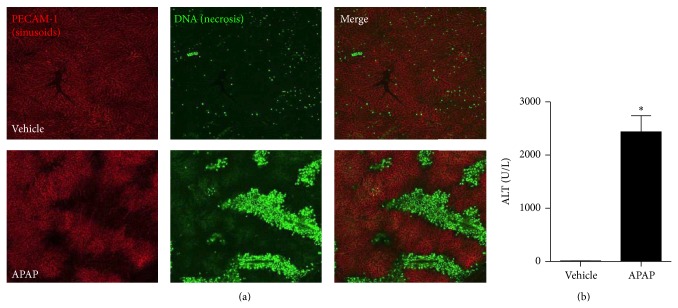
Acetaminophen model of acute liver injury and failure. (a) Liver confocal intravital microscopy showing that while control mice have a well perfused liver vasculature (evidenced by PE-anti-CD31 antibody staining, in red) and absence of necrosis (in green), APAP-treated mice (600 mg/Kg) had several dark areas in the red channel, which are suggestive of malperfusion caused by necrosis (elicited by DNA staining using Sytox Green). (b) Serum liver transaminase levels confirmed necrosis.  ^∗^Statistical significance in comparison to controls (*P* < 0.05). A 10x objective was used during imaging. One-way ANOVA with Tukey's post hoc test. Data are expressed as mean ± S.E.M (*n* = 6 mice/treatment).

**Figure 5:**
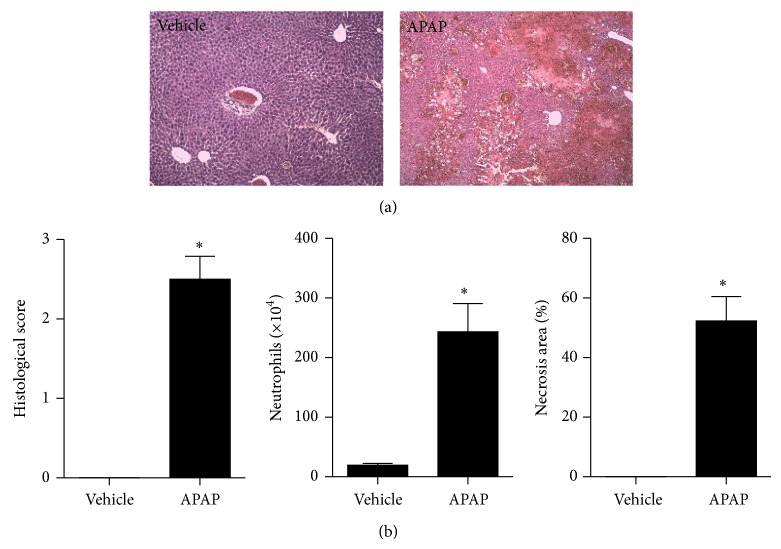
Histopathological assessment of acetaminophen-induced liver injury (APAP). (a) Liver histology (H&E) showing that while control mice have a healthy liver architecture and absence of necrosis, APAP-treated mice had several necrotic areas with leukocyte infiltration (green arrow). (b) Digital quantification of necrotic areas using ImageJ, histopathological score, and neutrophil infiltration (assessed by MPO levels) confirmed severe liver inflammation and necrosis induced by cocaine.  ^∗^Statistical significance in comparison to controls (*P* < 0.05). One-way ANOVA with Tukey's post hoc test. Data are expressed as mean ± S.E.M (*n* = 6 mice/treatment).

**Figure 6:**
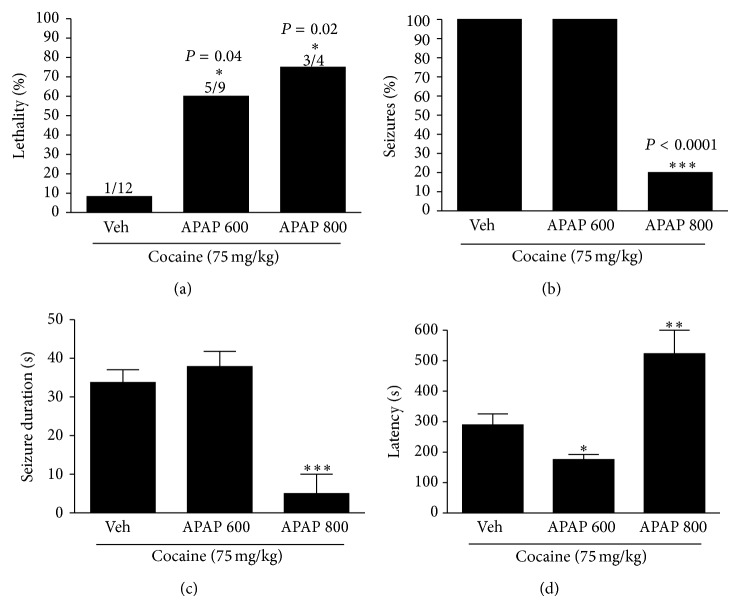
Previous hepatic injury (24 hours) with APAP interferes in seizure dynamics. (a) Administration of APAP at doses of 600 and 800 mg/kg 24 hours before cocaine administration increased lethality when compared to group vehicle/cocaine. (b) APAP at dose of 800 mg/kg reduced percentage of seizures and its duration (c). (d) Previous administration of APAP at dose of 600 mg/kg decreased seizure latency. APAP at dose of 800 mg/kg enhanced seizure latency.  ^∗^Significantly different from vehicle group (^∗^ *P* < 0.05).  ^∗∗^Significantly different from vehicle group (^∗∗^ *P* < 0.001).  ^∗∗∗^Significantly different from vehicle group (^∗∗∗^ *P* < 0.0001). One-way ANOVA with Tukey's post hoc test. Data are expressed as mean ± S.E.M (*n* = 6 mice/treatment).

In order to elucidate the relationship between liver injury and seizures, we first studied the dynamics of seizure due to cocaine overdose. Following a single cocaine injection, seizure was observed in all animals, manifested as rapid myoclonic convulsions of forelimbs and tail clonus preceded by locomotor depression (Figure 3(a)), as demonstrated by reduced seizure latency and long lasting seizure (Figure 3(b)). Interestingly, we observed a direct correlation between liver injury and function with seizure duration, suggesting that reduced liver metabolism might predispose to more severe seizure due to cocaine intake (Figure 3(c)). This is particularly relevant in the clinics because it might suggest that patients with previous liver damage are more susceptible to convulsive seizure during cocaine overdose. In this direction, we next investigated if a previous liver injury caused by another hepatotoxic drug worsens seizure profile induced by cocaine. For this, we established a model of hepatic injury due to acetaminophen (APAP; 600 mg/Kg) overdose. As shown in Figure 4(a), APAP caused extensive liver injury following 24 hs of overdose, and overt DNA deposition (in green) was observed in nonperfused areas (black areas in the red channel). Elevated levels of serum ALT (Figure 4(b)) and histopathology examination confirmed acute liver injury (Figure 5(a)), which occurred concomitantly to hepatic neutrophil infiltration (Figure 5(b)). APAP-treated mice that were challenged with a single dose of cocaine had significantly higher lethality in comparison to cocaine alone (Figure 6(a)). However, mice treated with higher doses of APAP had lower seizure incidence (Figure 6(b)) and duration (Figure 6(c)), with higher latency (Figure 6(d)), suggesting that metabolic brain disorders induced by acute liver failure can interfere in seizure dynamics, reinforcing the direct correlation of liver metabolic status with brain function.

### 3.3. Pretreatment with Cannabidiol (CBD) Reduces Cocaine-Induced Liver Injury and Seizure

**Figure 7:**
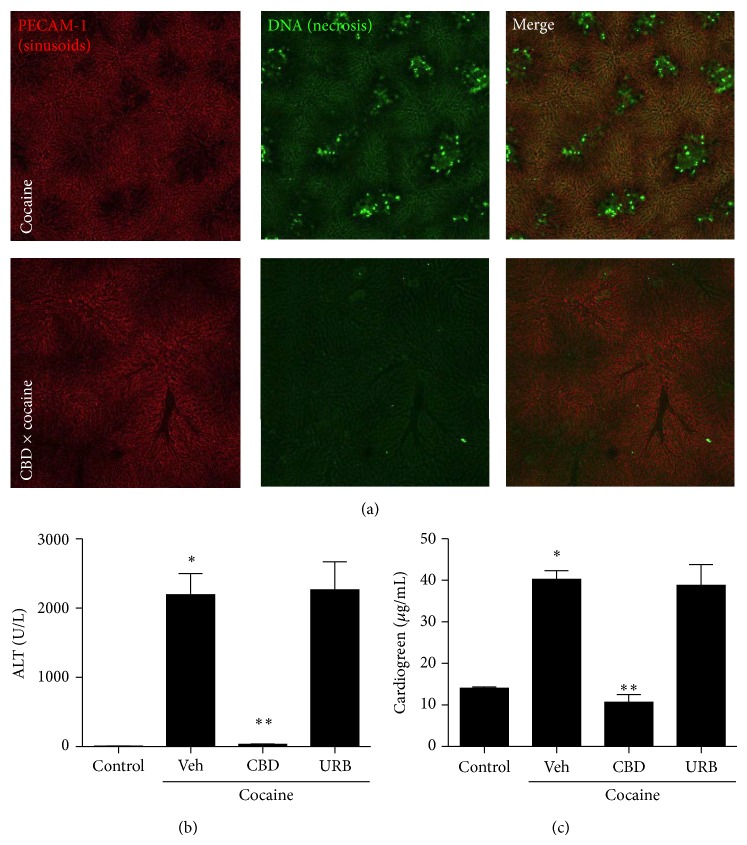
Cannabidiol (CBD; 30 mg/Kg) prevented cocaine-induced liver injury. (a) Liver confocal intravital microscopy showing that while CBD-treated mice have a well perfused liver vasculature (evidenced by PE-anti-CD31 antibody staining, in red) and absence of necrosis (in green), cocaine-treated mice (75 mg/Kg) had several dark areas in the red channel, which are suggestive of malperfusion caused by necrosis (elicited by DNA staining using Sytox Green). (b) Reduced serum liver transaminase levels and (c) clearance of cardiogreen confirmed inhibition of cocaine-induced liver injury by CBD. However, inhibition of anandamide hydrolysis by URB had no detectable effect.  ^∗^Statistical significance in comparison to controls (*P* < 0.05) and  ^∗∗^in comparison to vehicle-treated group. A 10x objective was used during imaging. One-way ANOVA with Tukey's post hoc test. Data are expressed as mean ± S.E.M (*n* = 6 mice/treatment).

**Figure 8:**
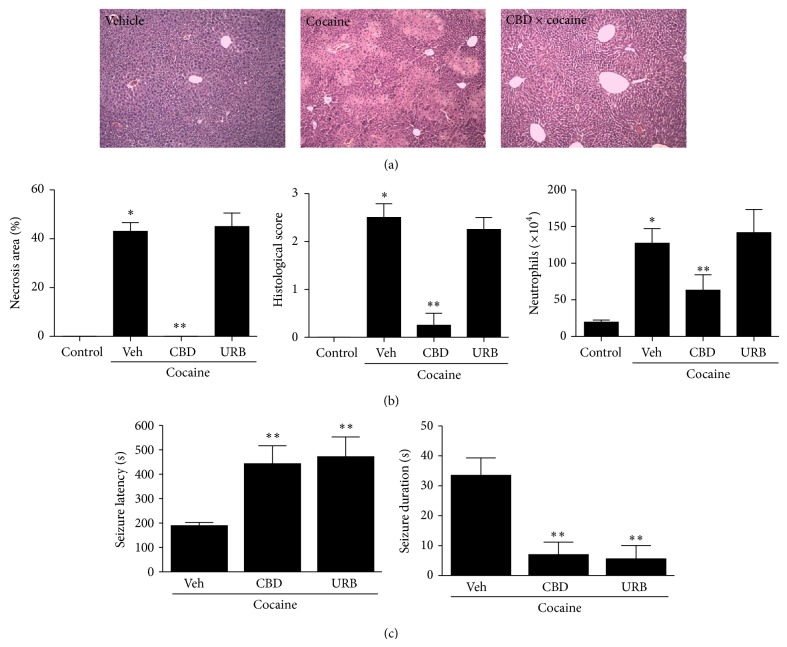
Cannabidiol (CBD; 30 mg/Kg) prevented liver inflammation and seizure due to cocaine intake. (a) Liver histology showing that while CBD-treated mice have a normal liver architecture, cocaine-treated mice (75 mg/Kg) had extensive areas which were suggestive of malperfusion caused by necrosis. (b) Reduced necrotic area, histological score, and neutrophil infiltration confirmed the anti-inflammatory and protective effects of CBD during cocaine-induced hepatotoxicity. However, inhibition of anandamide hydrolysis by URB had no detectable effect on liver parameters. (c) Despite lack of effect on liver injury, both CBD and URB inhibited cocaine-induced seizure, increasing latency and reducing seizure duration.  ^∗^Statistical significance in comparison to controls (*P* < 0.05) and  ^∗∗^in comparison to vehicle-treated group. A 10x objective was used during imaging. One-way ANOVA with Tukey's post hoc test. Data are expressed as mean ± S.E.M (*n* = 6 mice/treatment).

It is well accepted that cannabinoids may have anti-inflammatory activities and had been proposed to treat neurological disorders. In this context, the use of nonpsychotropic molecules is preferable in comparison to the herb itself, which also offers the opportunity to dosage adjustments. Taking into account the relationship between hepatic inflammation and brain disorders, we next investigated the participation of the cannabinoid system in our model. For this, we pretreated mice with cannabidiol (CBD; 30 mg/Kg), a major nonpsychotomimetic phytocannabinoid. In fact, pretreatment with CBD completely rescued liver injury induced by cocaine, as assessed by reduced liver DNA deposition (Figure 7(a)), and reduced serum ALT (Figure 7(b)) and cardiogreen (Figure 7(c)) levels, which prevented hepatic dysfunction due to cocaine overdose. Histopathology analysis confirmed that CDB-treated mice had no detectable injury due to cocaine injection (Figure 8(a)), and lower liver inflammation (estimated by neutrophil infiltration) was also observed (Figure 8(b)). In line with this, CBD treatment also reduced cocaine-induced seizure duration and higher latency (Figure 8(c)). Taking into account the potent effects of CBD, we next investigated if the inhibition of degradation of endogenous endocannabinoids might mimic the beneficial effects of the administration of the exogenous compound (CBD). URB597 mimicked the anticonvulsant effects of CBD, and URB-treated mice had less severe seizure induced by cocaine (Figure 8(c)). However, pretreatment with URB caused no detectable effects of cocaine-induced liver injury, suggesting that despite its protective central effects on seizure dynamics, exogenous agonists and higher doses are necessary for reaching the hepatoprotective effects of cannabinoid system activation.

## 4. Discussion

In the present study we demonstrated that CBD inhibited cocaine-induced seizure and liver injury in mice, alleviating hepatic inflammatory process, and reduced cocaine lethality. The FAAH inhibitor, URB597, inhibited seizure but did not interfere with hepatic inflammatory process. We also observed that previous hepatic dysfunction caused by APAP increased cocaine-induced seizure and lethality.

Drug abuse and addiction constitute a public health problem of great importance with a high prevalence worldwide. According to the United Nations Office on Drugs and Crime (UNODC, 2013), estimated number of cocaine users globally ranges between 14 and 21 million (0.3–0.5% of the population aged between 15 and 64 years) [42]. Acute cocaine abuse may induce several neurological impairments, including seizures and fulminant hepatic failure. Since there are no effective treatments available, it is associated with severe intoxications and deaths [3, 43]. Thus, our results showed that cannabidiol displays neuroprotective and hepatoprotective effects against cocaine toxicity and therefore might be used as future strategy in clinical emergency. Although our data provide support for the efficacy of CBD in this condition, careful pharmacologic studies are needed to further delineate specific mechanisms of CBD against cocaine toxicity.

The mechanisms involved in cocaine intoxication and seizure induction have remained unclear. Its psychotomimetic and psychostimulant effects are mediated by blockade of dopamine, serotonin, and norepinephrine reuptake. The increased stimulation of dopamine D2 receptors may also contribute to cocaine-induced seizures [44]. Clinical [45–47] and experimental [33, 48–51] observations demonstrate that the acute administration of high doses of this drug promotes convulsive seizures and liver abnormalities or fulminant hepatic failure. Less attention, however, has been directed towards possible hepatotoxic effects [3]. This drug induces increase in ALT activity and pervasive centrilobular foci of necrosis crowded with pale and swollen hepatocytes [52]. Several case reports have described hepatic injury from cocaine use, including cases in which cocaine-induced liver damage resulted in mortality [44].

Here, we described for the first time that previous treatment with phytocannabinoid CBD abolished cocaine induced acute seizure in mice, indicating anticonvulsant effect, in parallel with anti-inflammatory effect and protection against liver injury. CBD is the major nonpsychoactive component of cannabis that exerts multiple pharmacological actions in the central nervous system and in the periphery, besides being well-tolerated and exhibiting a broad spectrum of therapeutic properties [53]. Thus, it is currently attracting considerable interest as a potential medicine due to its anti-inflammatory, neuroprotective, antipsychotic, anxiolytic, antiepileptic, and anticancer effects [12, 17, 54].

One of the main actions of CBD is its anticonvulsant effect. In many experimental models, it exerted antiseizure activities against PTZ-induced generalized seizures in rats [55], in the pilocarpine model temporal lobe seizures and the penicillin model of partial seizures [56] as well as in the PTZ- and electroshock-induced seizures in rats [57]. Thus, considering the efficacy of CBD and the fact that it does not share the psychotomimetic, amnestic, and sedative effects of Δ^9^-THC, it is an interesting potential treatment for epilepsies. A recently clinical study observed that CBD-enriched cannabis extracts significantly reduced seizure frequency in children with treatment-resistant epilepsy, indicating that it could be used as a strategy when other pharmacological treatments fail [18].

In addition, the mechanisms of action of this compound have remained elusive and should be further investigated. CBD is a compound with a wide plethora of pharmacological actions, including anti-inflammatory and antioxidative effects, which may act through diverse mechanisms, facilitating the endocannabinoid system by blocker FAAH enzyme, binding to TRPV1 channels, serotonin type-1A receptors, A2A adenosine receptors, and peroxisome proliferator-activated receptors (i.e., PPAR-*γ*) among others [15, 17, 58]. In most in vivo models of inflammation, CBD attenuates inflammatory cell migration/infiltration (e.g., neutrophils) [59]. CBD suppresses T cell responses, decreases the release of bioactive tumour necrosis factor (TNF*α*), and reduces prostaglandin E2 (PGE2) levels, cyclooxygenase (COX) activity, and production of nitric oxide (NO). The suppressive effects of CBD on cellular immune responses and on the production of proinflammatory mediators may indicate its usefulness in several inflammatory diseases [17]. Additionally, CBD administration was able to decrease leukocyte migration into the lungs, myeloperoxidase activity in the lung tissue, protein concentration and production of proinflammatory cytokines (TNF*α* and IL-6), and chemokines (MCP-1 and MIP-2) in the bronchoalveolar lavage supernatant showing a potent anti-inflammatory effect in mice submitted to LPS-induced acute lung injury [60]. In other context, CBD modified the deleterious effects of inflammation in a viral model of multiple sclerosis through decreasing the transmigration of blood leukocytes by downregulating the expression of vascular cell adhesion molecule-1 (VCAM-1), chemokines (CCL2 and CCL5), and the proinflammatory cytokine IL-1*β* [61].

In respect to effects of CBD on liver, recent data showed that CBD protected against liver toxicity induced by a single dose of cadmium chloride [62]. Similarly, CBD attenuated the deterioration in the measured biochemical parameters and damages mediated by ischemia/reperfusion liver injury besides reducing the inflammatory response in tissue liver [24]. Previous studies already revealed that CBD pretreatment significantly protected against liver ischemia for 60 min followed by reperfusion for 24 hs [25].

Finally, our results on peripheric and central effects of CBD against toxic effects of cocaine are in agreement with previous results showing that this compound restored both liver and brain function in a model of hepatic encephalopathy associated with fulminant hepatic failure induced in mice by thioacetamide [26]. Pretreatment with CBD was able to revert neurological and cognitive deterioration induced by thioacetamide in parallel with increases of liver function, confirmed by improve of ammonia, bilirubin, aspartate transaminase (AST), and alanine transaminase (ALT) levels. These results showed that CBD effects might result from a combination of its actions in the liver and brain.

In conclusion, the present study demonstrates that cocaine-induced seizures occur along with severe hepatic toxicity. The phytocannabinoid, CBD, but not the anandamide hydrolysis inhibitor, URB597, protects against both effects and warrants further investigation as a potential treatment for both brain and liver consequences of cocaine intoxication.
